# Supplementary material for: Phase I trial of the TNF-α inhibitor certolizumab plus chemotherapy in stage IV lung adenocarcinomas
Source: Nat Commun. 2022 Oct 15;13:6095. doi: 10.1038/s41467-022-33719-6 (PMC9568581; doi:10.1038/s41467-022-33719-6)
Supplement: Supplementary file 1 — Supplementary Information [file 41467_2022_33719_MOESM1_ESM.pdf]

Supplementary Figure 1

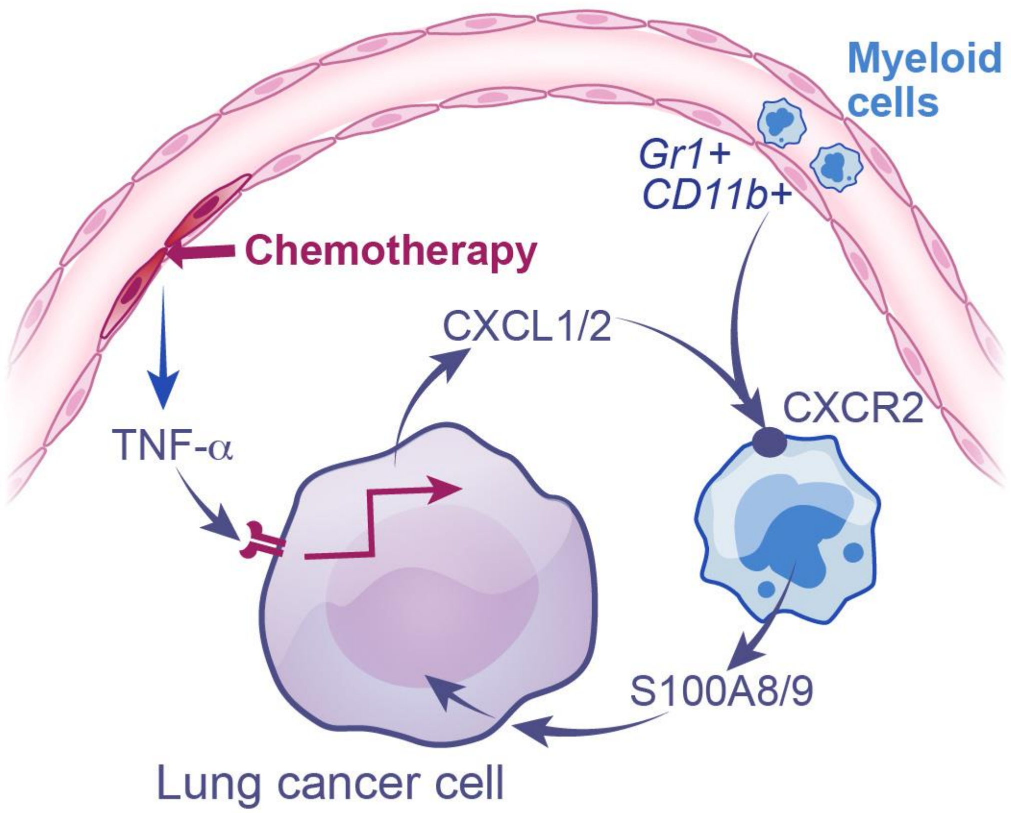

Supplementary Figure 1. Model of TNF-α mediated paracrine inflammatory loop that engenders chemotherapy resistance and metastasis propagation delineated in Acharyya et al. Cell 150:1 (165-178), 2012. Cytotoxic chemotherapy induces TNF-α from endothelial cells, which enhances CXCL1/2 production in cancer cells. CXCL1/2, the ligand for CXCR2, recruits Gr1+CD11b+ myeloid derived suppressor cells to the tumor microenvironment wherein production of S100A8/9 enhances cancer cell viability and triggers metastasis propagation.

Supplementary Figure 2

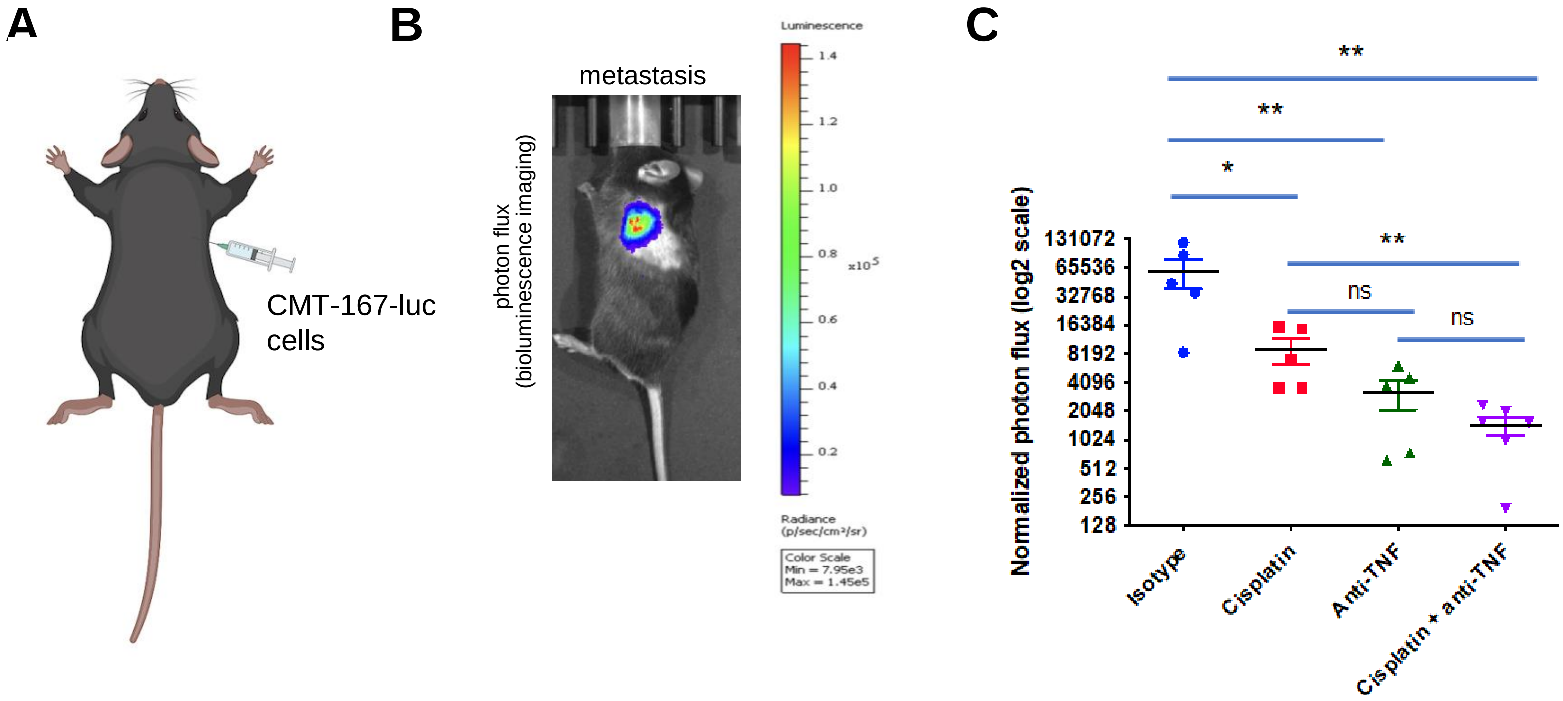

Supplementary Figure 2. Anti-TNF-α antibodies enhance the anti-tumor effect of cisplatin in a syngeneic lung orthotopic model. A-B. Schematic showing lung orthotopic injections (A) and lung photon flux by bioluminescence imaging [Created with BioRender.com] (B). C. For the orthotopic lung model, 1x10^5 murine CMT-167 lung cancer cells were injected into the lung of 8-to-9-week-old syngeneic C57BL/6 male mice. CMT-167 primarily showed tumors in the lung and occasionally showed tumors in the chest cavity. Mice were monitored by bioluminescence imaging. Mean normalized photon flux bioluminescence levels following treatment with either vehicle, cisplatin, anti-TNF, or cisplatin + anti-TNF are indicated for each group (biologically independent mice per group - n=5: isotype, cisplatin, anti-TNF; n=6 cisplatin + anti-TNF). Source data are provided as a Source Data file. Values are mean ± standard error. *p=0.0317, **p=0.0043, ns=not significant. Data are representative of two independent experiments.

Supplementary Figure 3

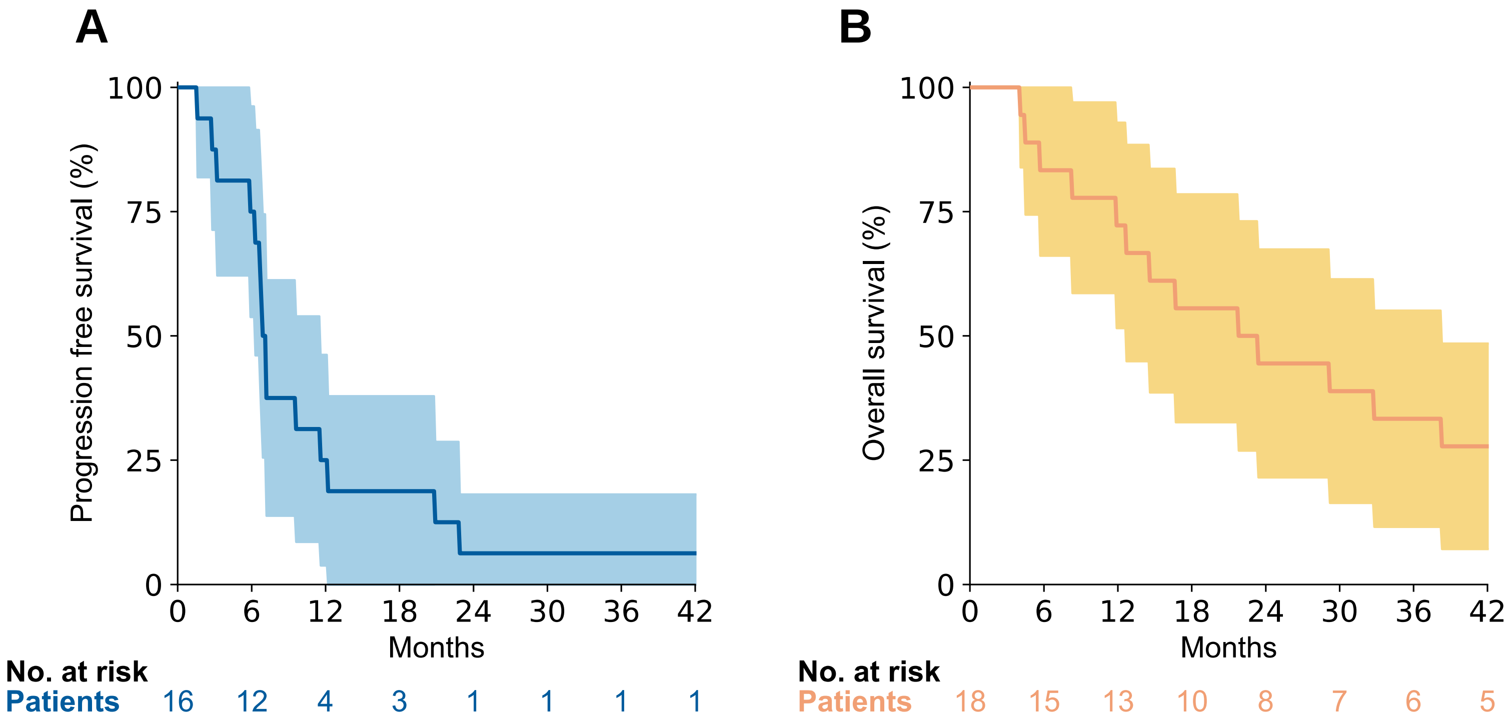

Supplementary Figure 3. Kaplan-Meier estimates of A. progression-free survival ([PFS] median PFS 7.1 months, 95% CI 6.30 to NR) and B. overall survival (Median overall survival 22.6 months, 95% CI 12.7 to NR). Bands depict vertical 95% CI. Source data are provided as a Source Data file.

Supplementary Figure 4

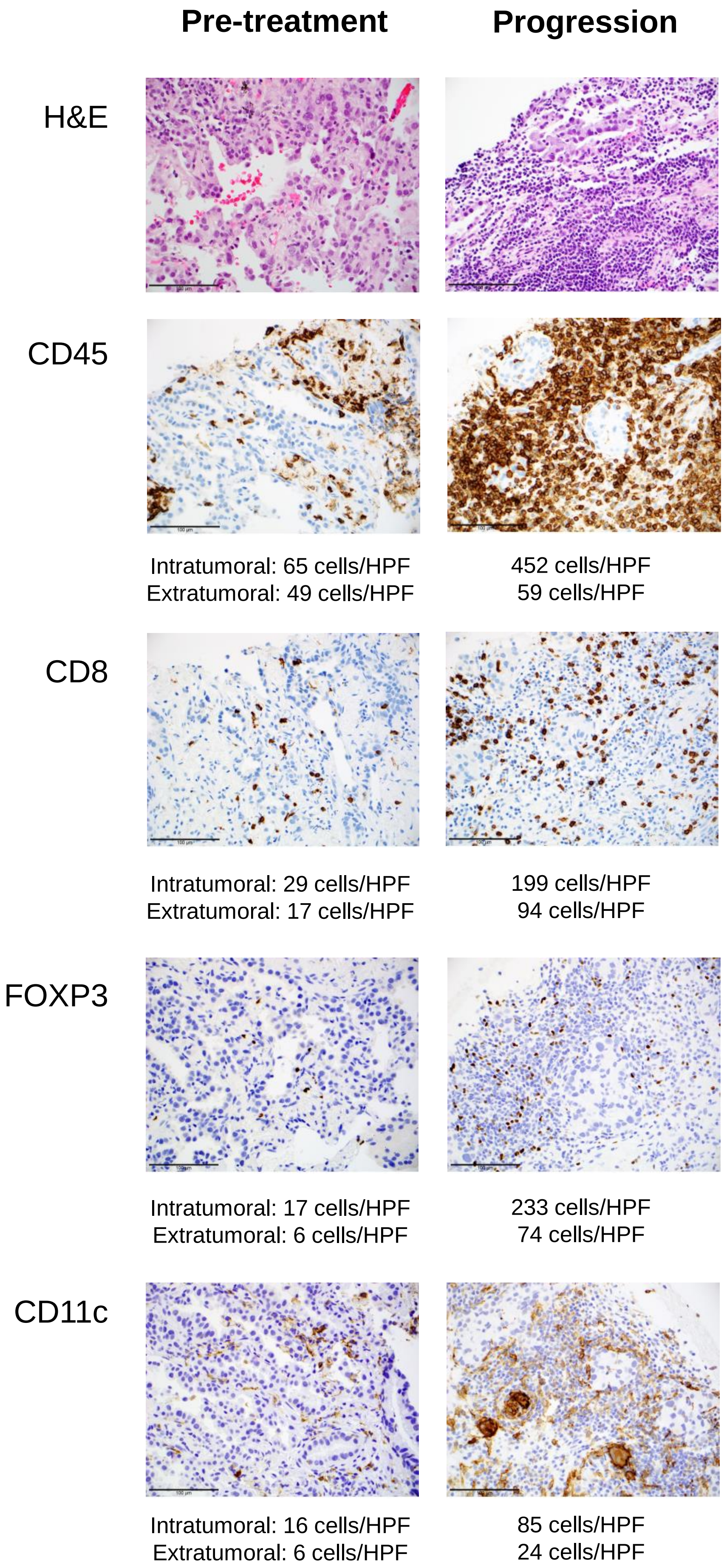

Supplementary Figure 4. H&E and immunohistochemistry images (antibodies staining for CD45, CD8, FOXP3, and CD11c) of patient 3, whose tumor showed a significant increase in immune cell infiltration after treatment. Scale bars denotes 100 μm.

Supplementary Table 1. Patient genotype table

Certolizumab group

Patient ID	Diagnostic Assay	Results
1	PCR; ALK FISH	Negative
2	IMPACT	POSITIVE FOR THE FOLLOWING SOMATIC ALTERATIONS IN THE CLINICALLY VALIDATED PANEL: 1. KRAS (NM_033360) exon2 p.G12V (c.35G>T) POSITIVE FOR THE FOLLOWING SOMATIC ALTERATIONS IN THE INVESTIGATIONAL PANEL: 2. CCND2 (NM_001759) exon5 p.L255F (c.763C>T) 3. ERCC2 (NM_000400) exon7 p.Q187H (c.560_561delinsGT) 4. SMAD4 (NM_005359) exon7 p.Q289X (c.865C>T) 5. STK11 (NM_000455) exon5 splicing variant (c.733C>A)
3	PCR; ALK FISH	KRAS G12D
4	N/A	N/A
5	IMPACT	POSITIVE FOR THE FOLLOWING SOMATIC ALTERATIONS IN THE INVESTIGATIONAL PANEL: 1. SDHA (NM_004168 - 5p15.33) Amplification (Fold Change: 2.2) 2. ATM (NM_000051) exon34 p.G1676L (c.5026_5027delinsTT) 3. BRIP1 (NM_032043) exon19 p.S927G (c.2779A>G) 4. CARD11 (NM_032415) exon21 p.R932L (c.2795G>T) 5. CREBBP (NM_004380) exon2 p.G252C (c.754G>T) 6. DICER1 (NM_030621) exon23 p.K1363N (c.4089G>T) 7. DICER1 (NM_030621) exon24 p.W1429L (c.4286G>T) 8. EPHA5 (NM_004439) exon8 p.A565S (c.1693G>T) 9. ERG (NM_182918) exon3 p.G95fs (c.284delG) 10. FLT1 (NM_002019) exon5 splicing variant (c.389-1G>C) 11. GRIN2A (NM_001134407) exon10 p.Q710H (c.2130G>T) 12. JAK3 (NM_000215) exon2 p.V28L (c.82G>T) 13. MPL (NM_005373) exon2 p.E54X (c.160G>T) 14. NOTCH4 (NM_004557) exon21 p.C1125X (c.3375C>A) 15. PTPRT (NM_133170) exon29 p.R1343W (c.4027C>T) 16. SMAD4 (NM_005359) exon9 p.E330K (c.988G>A) 17. TSC2 (NM_000548) exon23 p.Y865C (c.2594A>G)
6	PCR; ALK FISH	Negative
7	IMPACT	POSITIVE FOR THE FOLLOWING SOMATIC ALTERATIONS IN THE CLINICALLY VALIDATED PANEL: 1. TP53 (NM_000546) exon4 p.D49V (c.146A>T) POSITIVE FOR THE FOLLOWING SOMATIC ALTERATIONS IN THE INVESTIGATIONAL PANEL: 2. EGFR (NM_005228 - 7p11.2) Gain (Fold Change: 1.5) 3. PLK2 (NM_006622) exon10 p.I445M (c.1335T>G) 4. EGFR (NM_005228) rearrangement : c.2061+100_3114+140dup see Note1. Note1: The EGFR (NM_005228) rearrangement results in the duplication of exons 18 to 25 of EGFR, resulting in kinase domain duplication (see PMID: 26286086).

8	IMPACT	POSITIVE FOR THE FOLLOWING SOMATIC ALTERATIONS IN THE CLINICALLY VALIDATED PANEL: 1. EGFR (NM_005228) exon18 p.S720F (c.2159C>T) 2. EGFR (NM_005228) exon21 p.L861Q (c.2582T>A) 3. TP53 (NM_000546) exon6 p.H193P (c.578A>C) POSITIVE FOR THE FOLLOWING SOMATIC ALTERATIONS IN THE INVESTIGATIONAL PANEL: 4. AKT1 (NM_001014431 - 14q32.33) Amplification (Fold Change: 2.1)
9	IMPACT	POSITIVE FOR THE FOLLOWING SOMATIC ALTERATIONS IN THE CLINICALLY VALIDATED PANEL: 1. KRAS (NM_033360) exon2 p.G12C (c.34G>T) 2. TP53 (NM_000546) exon5 p.K132R (c.395A>G) POSITIVE FOR THE FOLLOWING SOMATIC ALTERATIONS IN THE INVESTIGATIONAL PANEL: 3. ARID2 (NM_152641) exon14 p.Q613* (c.1837C>T) 4. ASXL1 (NM_015338) exon12 p.R718I (c.2153G>T) 5. CARD11 (NM_032415) exon13 p.P568Q (c.1703C>A) 6. EPHA5 (NM_004439) exon16 splicing variant (c.2658+1G>T) 7. EPHA7 (NM_004440) exon17 p.S980R (c.2940C>A) 8. INHA (NM_002191) exon1 p.G42W (c.124G>T) 9. INPP4B (NM_001101669) exon18 p.P572A (c.1714C>G) 10. NCOR1 (NM_006311) exon15 splicing variant (c.1408-1G>C) 11. NCOR1 (NM_006311) exon14 p.E485D (c.1455G>C) 12. NF1 (NM_001042492) exon3 p.R69G (c.205A>G) 13. PTPRD (NM_002839) exon15 p.Q133K (c.397C>A)
10	PCR; ALK FISH	Negative
13	IMPACT	POSITIVE FOR THE FOLLOWING SOMATIC ALTERATIONS IN THE INVESTIGATIONAL PANEL: 1. CDKN2B (NM_004936 - 9p21.3) Deletion (Fold Change: -2.0) 2. CDKN2Ap16INK4A (NM_000077 - 9p21.3) Deletion (Fold Change: -2.0) 3. CDKN2Ap14ARF (NM_058195 - 9p21.3) Deletion (Fold Change: -2.0) 4. EML4 (NM_001145076) - ALK (NM_004304) Fusion (EML4 exon 13 fused with ALK exon 20) : c.2068+156:EML4_c.3173-693:ALKinv
11	IMPACT	POSITIVE FOR THE FOLLOWING SOMATIC ALTERATIONS IN THE CLINICALLY VALIDATED PANEL: 1. KRAS (NM_033360) exon2 p.G12A (c.35G>C) POSITIVE FOR THE FOLLOWING SOMATIC ALTERATIONS IN THE INVESTIGATIONAL PANEL: 2. NFKBIA (NM_020529 - 14q13.2) Gain (Fold Change: 1.8) 3. NKX2-1 (NM_001079668 - 14q13.3) Gain (Fold Change: 1.8) 4. ATM (NM_000051) exon6 p.Q218* (c.652C>T) 5. BCL6 (NM_001706) exon5 p.S444I (c.1331G>T) 6. FUBP1 (NM_003902) exon3 p.K77N (c.231A>T) 7. MED12 (NM_005120) exon10 p.G454V (c.1361G>T) 8. PDGFRB (NM_002609) exon12 p.R561P (c.1682G>C) 9. SPEN (NM_015001) exon11 p.P2434L (c.7301C>T) 10. ZFHX3 (NM_006885) exon3 p.G919C (c.2755G>T)

12	IMPACT	POSITIVE FOR THE FOLLOWING SOMATIC ALTERATIONS IN THE INVESTIGATIONAL PANEL: 1. MYC (NM_002467 - 8q24.21) Amplification (Fold Change: 4.2) 2. MDM2 (NM_002392 - 12q15) Amplification (Fold Change: 2.5) 3. CDKN1B (NM_004064 - 12p13.1) Amplification (Fold Change: 2.1) 4. CDKN2B (NM_004936 - 9p21.3) Deletion (Fold Change: -2.3) 5. CDKN2Ap14ARF (NM_058195 - 9p21.3) Deletion (Fold Change: -2.3) 6. CDKN2Ap16INK4A (NM_000077 - 9p21.3) Loss (Fold Change: -1.8) 7. BIRC3 (NM_182962) exon3 p.E216K (c.646G>A) 8. CSF3R (NM_000760) exon6 p.E206Q (c.616G>C) 9. FLT3 (NM_004119) exon18 p.Q744* (c.2230C>T) 10. IRS2 (NM_003749) exon1 p.R301W (c.901C>T) 11. JAK2 (NM_004972) exon13 p.V567E (c.1700T>A) 12. KEAP1 (NM_203500) exon2 p.G186V (c.557G>T) 13. RAD50 (NM_005732) exon8 p.R352H (c.1055G>A) 14. STK11 (NM_000455) exon5 splicing variant (c.598-2A>T) 15. TET2 (NM_001127208) exon3 p.R369W (c.1105C>T)
14	N/A	N/A
15	IMPACT	POSITIVE FOR THE FOLLOWING SOMATIC ALTERATIONS IN THE CLINICALLY VALIDATED PANEL: 1. KRAS (NM_033360) exon2 p.G12D (c.35G>A) POSITIVE FOR THE FOLLOWING SOMATIC ALTERATIONS IN THE INVESTIGATIONAL PANEL: 2. EPHA7 (NM_004440) exon3 p.S251R (c.753T>A) 3. MAX (NM_002382) exon6 splicing variant (c.296-1G>T) 4. MLL2 (NM_003482) exon39 p.T4332A (c.12994A>G)
16	IMPACT	POSITIVE FOR THE FOLLOWING SOMATIC ALTERATIONS IN THE CLINICALLY VALIDATED PANEL: 1. TP53 (NM_000546) exon5 p.R175H (c.524G>A) POSITIVE FOR THE FOLLOWING SOMATIC ALTERATIONS IN THE INVESTIGATIONAL PANEL: 2. BARD1 (NM_000465) exon7 p.D535V (c.1604A>T) 3. BCOR (NM_001123385) exon2 p.S3L (c.8C>T) 4. BRCA2 (NM_000059) exon25 p.E3157Q (c.9469G>C) 5. CD79B (NM_001039933) exon4 p.Q154K (c.460C>A) 6. CDK12 (NM_016507) exon1 p.R33T (c.98G>C) 7. CDKN1A (NM_078467) exon3 p.R94P (c.281G>C) 8. CIC (NM_015125) exon14 p.S1105* (c.3314C>G) 9. CRLF2 (NM_022148) exon4 p.V136L (c.406G>T) 10. DIS3 (NM_014953) exon15 p.I649V (c.1945A>G) 11. EPHA7 (NM_004440) exon4 p.G280C (c.838G>T) 12. EPHB1 (NM_004441) exon5 p.A398G (c.1193C>G) 13. EPHB1 (NM_004441) exon13 p.E794D (c.2382G>T) 14. ERBB3 (NM_001982) exon8 p.D297H (c.889G>C) 15. ERBB4 (NM_005235) exon28 p.L1163V (c.3487C>G) 16. EZH2 (NM_004456) exon4 p.K99fs (c.295_296delinsG) 17. FGFR4 (NM_213647) exon7 p.G264V (c.791G>T) 18. FLT3 (NM_004119) exon24 p.Q987L (c.2960A>T) 19. FOXP1 (NM_001244814) exon6 p.Q211H (c.633G>T) 20. GLI1 (NM_005269) exon12 p.Q867K (c.2599C>A) 21. HOXB13 (NM_006361) exon1 p.G176V (c.527G>T) 22. IKZF1 (NM_006060) exon7 p.E259Q (c.775G>C) 23. IRS2 (NM_003749) exon1 p.S342W (c.1025C>G) 24. KEAP1 (NM_203500) exon6 splicing variant (c.1532-2A>G) 25. MDC1 (NM_014641) exon8 p.G878V (c.2633G>T) 26. NCOA3 (NM_181659) exon7 p.M217I (c.651G>T) 27. PALB2 (NM_024675) exon4 p.A188S (c.562G>T) 28. PALB2 (NM_024675) exon4 p.T274S (c.820A>T) 29. PARP1 (NM_001618) exon1 p.E26Q (c.76G>C) 30. PIK3R2 (NM_005027) exon16 p.T673M (c.2018C>T) 31. PTPRD (NM_002839) exon39 p.P1520T (c.4558C>A) 32. PTPRT (NM_133170) exon2 p.S39R (c.115A>C) 33. PTPRT (NM_133170) exon9 p.W506L (c.1517G>T) 34. SMARCA4 (NM_003072) exon30 p.D1432fs (c.4294delG) 35. SOX17 (NM_022454) exon2 p.E277D (c.831G>T) 36. TET1 (NM_030625) exon4 p.H845L (c.2534A>T) 37. ZFHX3 (NM_006885) exon9 p.S2098L (c.6293C>T) 38. CDKN2Ap16INK4A (NM_000077) rearrangement: c.150+436del_c.263del (Note1) 39. CDKN2Ap14ARF (NM_058195) rearrangement: c.194-3034_c.306del (Note2)

Control group

Patient ID	Diagnostic Assay	Results
MSK-024	PCR; ALK FISH	POSITIVE FOR BRAF MUTATION p.V600E (c.1799 T>A) No other mutations detected within this testing panel
MSK-025	IMPACT	POSITIVE FOR THE FOLLOWING SOMATIC ALTERATIONS IN THE CLINICALLY VALIDATED PANEL: 1. KRAS (NM_033360) exon2 p.G12C (c.34G>T) POSITIVE FOR THE FOLLOWING SOMATIC ALTERATIONS IN THE INVESTIGATIONAL PANEL: 2. CARM1 (NM_199141) exon1 p.P20T (c.58C>A) 3. EPHA5 (NM_004439) exon15 p.W844C (c.2532G>T) 4. KEAP1 (NM_203500) exon3 p.L281M (c.841C>A) 5. SOX17 (NM_022454) exon2 p.P330H (c.989C>A) 6. STK11 (NM_000455) exon7 p.L290R (c.869T>G)
MSK-026	IMPACT	POSITIVE FOR THE FOLLOWING SOMATIC ALTERATIONS IN THE CLINICALLY VALIDATED PANEL: 1. KRAS (NM_033360) exon2 p.G12C (c.34G>T) POSITIVE FOR THE FOLLOWING SOMATIC ALTERATIONS IN THE INVESTIGATIONAL PANEL: 2. AGO2 (NM_012154) exon12 p.E502Q (c.1504G>C) 3. ARID1A (NM_006015) exon15 p.S1248* (c.3743C>A) 4. ARID1A (NM_006015) exon20 p.R1879W (c.5635C>T) 5. ARID1A (NM_006015) exon20 p.S2096* (c.6287C>G) 6. ARID1A (NM_006015) exon20 p.S2264L (c.6791C>T) 7. CRKL (NM_005207) exon2 p.A255S (c.763G>T) 8. EPHA3 (NM_005233) exon16 p.V935L (c.2803G>T) 9. FLT4 (NM_182925) exon6 p.C252* (c.756C>A) 10. MLH1 (NM_000249) exon1 p.E34Q (c.100G>C) 11. PAK7 (NM_177990) exon4 p.S246F (c.737C>T) 12. RB1 (NM_000321) exon3 splicing variant p.X89_splice (c.265-1G>T) 13. TP53 (NM_000546) exon9 p.Q317* (c.949C>T)
MSK-027	IMPACT	POSITIVE FOR THE FOLLOWING SOMATIC ALTERATIONS IN THE CLINICALLY VALIDATED PANEL: 1. KRAS (NM_033360) exon2 p.G12C (c.34G>T) POSITIVE FOR THE FOLLOWING SOMATIC ALTERATIONS IN THE INVESTIGATIONAL PANEL: 2. CIC (NM_015125) exon1 p.M21I (c.63G>T) 3. DICER1 (NM_030621) exon24 p.D1522H (c.4564G>C) 4. ERCC5 (NM_000123) exon8 p.A627S (c.1879G>T) 5. MAX (NM_002382) exon3 p.R33P (c.98G>C) 6. MITF (NM_198159) exon2 p.K79N (c.237G>T) 7. MTOR (NM_004958) exon43 p.T1977R (c.5930C>G) 8. PPP4R2 (NM_174907) exon8 p.R290H (c.869G>A) 9. RBM10 (NM_001204468) exon17 splicing variant p.X715_splice (c.2145+1G>T) 10. SOX17 (NM_022454) exon2 p.R125H (c.374G>A) 11. ZFHX3 (NM_006885) exon2 p.F590V (c.1768T>G)

MSK-028	IMPACT	POSITIVE FOR THE FOLLOWING SOMATIC ALTERATIONS IN THE CLINICALLY VALIDATED PANEL: 1. TP53 (NM_000546) exon5 p.N131I (c.392A>T) POSITIVE FOR THE FOLLOWING SOMATIC ALTERATIONS IN THE INVESTIGATIONAL PANEL: 2. FGFR1 (NM_001174067 - 8p11.22) Amplification (Fold Change: 2.4) 3. ALK (NM_004304) exon18 p.H976Qfs*6 (c.2928_2930delinsA) 4. ARID1A (NM_006015) exon20 p.Q2070Sfs*65 (c.6207delG) 5. AXL (NM_021913) exon4 p.T154S (c.460A>T) 6. CDKN2Ap16INK4A (NM_000077) exon2 p.M53T (c.158T>C) 7. DROSHA (NM_013235) exon4 p.P67S (c.199C>T) 8. HIST1H2BD (NM_021063) exon1 p.Y38C (c.113A>G) 9. INHBA (NM_002192) exon3 p.G275S (c.823G>A) 10. IRS2 (NM_003749) exon1 p.P954T (c.2860C>A) 11. KEAP1 (NM_203500) exon4 p.G509V (c.1526G>T) 12. MED12 (NM_005120) exon15 p.P699Q (c.2096C>A) 13. PREX2 (NM_024870) exon32 splicing variant p.X1318_splice (c.3954_3984+45del) 14. SMARCA4 (NM_003072) exon32 p.D1528Y (c.4582G>T) 15. STK11 (NM_000455) exon1 p.E70* (c.208G>T)
MSK-029	IMPACT	POSITIVE FOR THE FOLLOWING SOMATIC ALTERATIONS IN THE CLINICALLY VALIDATED PANEL: 1. TP53 (NM_000546) exon7 p.E258K (c.772G>A) POSITIVE FOR THE FOLLOWING SOMATIC ALTERATIONS IN THE INVESTIGATIONAL PANEL: 2. CD74 - ROS1 fusion (CD74 (NM_001025159) exons 1-7 fused to ROS1 (NM_002944) exons 32-43): t(5;6)(q32;q22.1)(chr5:g.149782871::chr6:g.117650623) 3. WHSC1L1 (NM_023034 - 8p11.23) Amplification (Fold Change: 2.1) 4. FGFR1 (NM_001174067 - 8p11.22) Amplification (Fold Change: 2.1) 5. CDKN2Ap14ARF (NM_058195) exon2 p.G102K (c.304_305delinsAA) 6. CDKN2Ap16INK4A (NM_000077) exon2 p.E88K (c.261_262delinsAA) 7. CTNNB1 (NM_001904) exon3 p.G34R (c.100G>A) 8. NOTCH4 (NM_004557) exon24 p.R1489Q (c.4466G>A) 9. RECQL (NM_032941) exon7 p.E205G (c.614A>G) 10. RPTOR (NM_020761) rearrangement: chr17:g.43042059_c.2298:RPTORdel (Note 1) Note 1: The RPTOR rearrangement is a deletion of exons 20-34. One of the breakpoints is within exon 20.
MSK-030	IMPACT	POSITIVE FOR THE FOLLOWING SOMATIC ALTERATIONS IN THE CLINICALLY VALIDATED PANEL: 1. KRAS (NM_033360) exon2 p.G12V (c.35G>T) 2. PIK3CA (NM_006218) exon10 p.E545K (c.1633G>A) 3. TP53 (NM_000546) exon7 p.R249S (c.747G>T) MICROSATELLITE STABLE (MSS). This result is for investigational use only. See MSI note below. POSITIVE FOR THE FOLLOWING SOMATIC ALTERATIONS IN THE INVESTIGATIONAL PANEL: 4. ATR (NM_001184) exon4 p.M197I (c.591G>A) 5. DNMT3A (NM_022552) exon14 splicing variant p.X519_splice (c.1555-1G>T) 6. KEAP1 (NM_203500) exon3 p.D236H (c.706G>C) 7. MTOR (NM_004958) exon38 p.S1777I (c.5330G>T) 8. PREX2 (NM_024870) exon13 p.C487* (c.1461T>A) 9. TP53 (NM_000546) exon11 splicing variant p.X367_splice (c.1101-2A>T) 10. XPO1 (NM_003400) exon15 p.E571K (c.1711G>A)

MSK-031	IMPACT	POSITIVE FOR THE FOLLOWING SOMATIC ALTERATIONS IN THE INVESTIGATIONAL PANEL: 1. YES1 (NM_005433 - 18p11.32) Amplification (Fold Change: 5.6) 2. APC (NM_000038) exon16 p.A2529L (c.7585_7586delinsTT) 3. ASXL2 (NM_018263) exon12 p.S1211C (c.3631A>T) 4. CALR (NM_004343) exon9 p.E381A (c.1142A>C) 5. EIF4A2 (NM_001967) exon8 p.T274K (c.821C>A) 6. FOXL2 (NM_023067) exon1 p.E69K (c.205G>A) 7. HIST1H3G (NM_003534) exon1 p.Q20* (c.58C>T) 8. INPP4B (NM_001101669) exon17 p.E520K (c.1558G>A) 9. MDC1 (NM_014641) exon8 p.T946S (c.2836A>T) 10. NF1 (NM_001042492) exon26 p.G1129Afs*13 (c.3384delT) 11. NF1 (NM_001042492) exon42 p.E2143* (c.6427G>T) 12. PDGFRA (NM_006206) exon3 p.G79C (c.235G>T) 13. STK11 (NM_000455) exon6 p.P281Rfs*6 (c.842delC) 14. TERT (NM_198253) promoter variant (g.1295228C>T) 15. WT1 (NM_024426) exon6 p.Y339* (c.1017C>A)
MSK-032	IMPACT	POSITIVE FOR THE FOLLOWING SOMATIC ALTERATIONS IN THE CLINICALLY VALIDATED PANEL: 1. TP53 (NM_000546) exon7 p.R249K (c.746G>A) POSITIVE FOR THE FOLLOWING SOMATIC ALTERATIONS IN THE INVESTIGATIONAL PANEL: 2. FAM58A (NM_152274 - Xq28) Amplification (Fold Change: 2.9) 3. PMAIP1 (NM_021127 - 18q21.32) Amplification (Fold Change: 2.0) 4. NFKBIA (NM_020529 - 14q13.2) Gain (Fold Change: 1.9) (Note 1) 5. NKX2-1 (NM_001079668 - 14q13.3) Gain (Fold Change: 1.9) (Note 1) 6. RTEL1 (NM_032957 - 20q13.33) Gain (Fold Change: 1.8) (Note 1) 7. TOP1 (NM_003286 - 20q12) Loss (Fold Change: -1.9) (Note 2) 8. PTPRT (NM_133170 - 20q13.11) Intragenic deletion 9. ERBB3 (NM_001982) exon28 p.M1292I (c.3876G>A) 10. KDR (NM_002253) exon13 p.V656F (c.1966G>T) 11. SDC4 (NM_002999) - ROS1 (NM_002944) fusion (SDC4 exons 1-2 fused in-frame to ROS1 exons 32-43): t(6;20)(q22.1;q13.12)(chr6:g.117654375::chr20:g.43963860) (Note 3) 12. NBN (NM_002485) rearrangement: g.90932931_c.994+81:NBNdel (Note 4) Note 1: The NFKBIA, NKX2-1, and RTEL1 copy number gains fall slightly below the cut off criteria for amplification. Confirmatory testing by an alternate method is suggested, if clinically indicated. Note 2: The TOP1 copy number loss falls slightly below the cut off criteria for deletion. Confirmatory testing by an alternate method is suggested, if clinically indicated. Note 3: The SDC4-ROS1 fusion is predicted to be in-frame and includes the kinase domain of ROS1.
MSK-033	N/A	N/A

IMPACT denotes next-generation sequencing by the MSK-IMPACT assay. ALK FISH denotes fluorescence in situ hybridization for the ALK gene.

PCR: polymerase chain reaction

Supplementary Table 2.

Association between PFS and TNF-α and S100A8 as time dependent covariates using a Cox proportional hazards regression model. HR is per ng/dL unit change for TNF-α, and per 1% change in S100A8 concentration.

	N	HR	P value
[TNF-α]	23	1.06	0.786
% change in S100A8	23	1.00	0.763

Association between OS and TNF-α and S100A8 as time dependent covariates using a Cox proportional hazards regression model.

	N	HR	P value
[TNF-α]	23	1.58	0.069
% change in S100A8	23	1.01	0.330

Supplementary Table 3. Tumor immune cell infiltration at baseline and upon progression by immunohistochemistry in 4 patients who received certolizumab

Patient ID	Certolizumab dose	Sample	Cells with + IHC staining/ hpf									
			CD45	CD3	CD4	CD8	CD56	CD11c	CD25	CD14	FOXp3	PD-L1
3	200mg	Right lung mass baseline										
		Intratumoral	65	62	N/A	29	1	16	4	14	17	0
		Extratumoral	49	38	N/A	17	1	6	1	0	6	N/A
		Right lung mass progression										
		Intratumoral	452	557	N/A	199	6	85	31	32	233	0
		Extratumoral	59	135	N/A	94	0	24	5	14	74	N/A
16	400mg	Left lung mass baseline										
		Intratumoral	44	52	0	34	1	11	2	11	19	70
		Extratumoral	N/A	N/A	N/A	N/A	N/A	N/A	N/A	N/A	N/A	N/A
		Left lung mass progression										
		Intratumoral	N/A	47	15	N/A	0	11	N/A	13	N/A	N/A
		Extratumoral	N/A	13	7	8	0	10	0	1	1	N/A
10	400mg	Left cervical node baseline										
		Intratumoral	0	34	0	28	1	21	2	0	4	99
		Extratumoral	N/A	N/A	N/A	N/A	N/A	N/A	N/A	N/A	N/A	N/A
		Right lung mass progression										
		Intratumoral	22	17	9	22	1	15	1	0	11	99
		Extratumoral	33	26	2	14	0	17	0	12	6	N/A
6	400mg	Subcutaneous nodule baseline										
		Intratumoral	0	12	3	4	0	24	3	6	7	10
		Extratumoral	0	6	3	9	1	2	2	9	13	N/A
		Lung mass progression										
		Intratumoral	5	17	2	6	1	6	1	3	6	5
		Extratumoral	2	8	3	3	0	7	0	4	2	N/A

Supplementary Table 4. Clinicopathologic characteristics for control group

	N=10
Adenocarcinoma	10 (100%)
Median age (range)	64 (53-79)
Stage	
IV	8 (80%)
IIIB	1 (10%)
IIA	1 (10%)
Female	40%
Smoking history	
Never	3 (30%)
Current/former	7 (70%)
Median pack years (range)	35 (0-60)
Median KPS (range)	90 (80-90)
Treatment regimens	
Carboplatin + pemetrexed + bevacizumab	6 (60%)
Cisplatin + pemetrexed	3 (30%)
Cisplatin + pemetrexed + bevacizumab	1 (10%)
